# Phytogenotypic Anthocyanin Profiles and Antioxidant Activity Variation in Fruit Samples of the American Cranberry (*Vaccinium macrocarpon* Aiton)

**DOI:** 10.3390/antiox11020250

**Published:** 2022-01-27

**Authors:** Rima Urbstaite, Lina Raudone, Valdimaras Janulis

**Affiliations:** 1 Department of Pharmacognosy, Faculty of Pharmacy, Lithuanian University of Health Sciences, 50166 Kaunas, Lithuania; lina.raudone@lsmuni.lt (L.R.); valdimaras.janulis@lsmuni.lt (V.J.); 2 Laboratory of Biopharmaceutical Research, Institute of Pharmaceutical Technologies, Lithuanian University of Health Sciences, 50166 Kaunas, Lithuania

**Keywords:** cranberry, anthocyanidin, antioxidant activity, UPLC, *Vaccinium macrocarpon*

## Abstract

In this study, we conducted an analysis of the qualitative and quantitative composition of anthocyanins and anthocyanidins in different cultivars and genetic clones of American cranberries grown in Lithuanian climatic conditions. Four anthocyanin compounds predominated in fruit samples of American cranberry cultivars: cyanidin-3-galactoside, cyanidin-3-arabinoside, peonidin-3-galactoside, and peonidin-3-arabinoside. They accounted for 91.66 ± 2.79% of the total amount of the identified anthocyanins. The total anthocyanin content detected via the pH differential method was found to be by about 1.6 times lower than that detected via the UPLC method. Hierarchical cluster analysis and principal component analysis showed that the ‘Woolman’ cultivar distinguished from other cranberry cultivars in that its samples contained two times the average total amount of anthocyanins (8.13 ± 0.09 mg/g). The group of American cranberry cultivars ‘Howes’, ‘Le Munyon’, and ‘BL-8’ was found to have higher than average levels of anthocyanidin galactosides (means 3.536 ± 0.05 mg/g), anthocyanidins (means 0.319 ± 0.01 mg/g), and total anthocyanins (means 6.549 ± 0.09 mg/g). The evaluation of the antioxidant effect of cranberry fruit sample extracts showed that the greatest radical scavenging activity of the cranberry fruit extracts was determined in the fruit samples of ‘Woolman’ (849.75 ± 10.88 µmol TE/g) and the greatest reducing activity was determined in ‘Le Munyon’ (528.05 ± 12.16 µmol TE/g). The study showed a correlation between the total anthocyanin content and the antiradical and reductive activity of the extracts in vitro (respectively, R = 0.635 and R = 0.507, *p* < 0.05).

## 1. Introduction

The American cranberry (*Vaccinium macrocarpon* Aiton) is a perennial evergreen plant of the *Ericaceae* A.L. de Jussie family growing in natural habitats in North America [1,2,3]. The selection of cranberry cultivars began in the U.S. in the early 1800s, in studies with cranberry plants growing in natural cenopopulations [4]. More than 200 cultivars of American cranberries are cultivated worldwide [5]. According to the data for 2019, the United States, Canada, and Chile provide 98% of world cranberry production [6].

In the climatic conditions of Lithuania, small cranberries (*Vaccinium oxycoccos* L.) grow in the natural cenopopulations of raised bogs and intermediate-type wetlands [7]. During the land reclamation works in Lithuania, those raised bogs and intermediate-type wetlands were drained, which decreased the areas of small cranberry habitats [7]. In about 1967, the selection and introduction of the first cultivars of American cranberries as a perennial berry culture began in Lithuania [8]. Recently, the cultivation of the introduced cranberry cultivars in Lithuania has gained popularity [7].

The most important groups of biologically active compounds found in cranberry fruits are flavonols (derivatives of quercetin and myricetin) [9], flavan-3-ols, anthocyanins [10], phenolic acids [11], and triterpenoids [12]. The cranberry-specific proantocyanidin and flavonol complex possess antiadhesive activity to the uropatogenic strains of *Escherichia coli* [13]. The effects of these biologically active compounds in cranberry fruit determine the use of the fruit in the prevention and treatment of urinary tract infections [14]. Studies on the effects of cranberry fruit extracts showed that quercetin in those extracts inhibited cell proliferation and reduced the growth of bladder [15] and ovarian [16] cancer cells [17].

Anthocyanins and anthocyanidins are one of the most important groups of biologically active compounds in cranberry fruit. Pappas and Schaich have found that in samples of freshly harvested cranberries, their levels could vary from 13.6 to 171 mg/100 g [18]. About 90% of the total amount of anthocyanins in cranberry samples are cyanidin and peonidin glycosides (cyanidin-3-arabinoside, cyanidin-3-galactoside, peonidine-3-arabinoside, and peonidine-3-galactoside) [19]. The other components of the anthocyanin complex in cranberry fruit make up a small percentage, amounting to about 10%. Of these, glycosides of delphinidin, cyanidin, petunidin, and malvidin are worth mentioning, as their molecules contain various monosaccharides [19].

Yan et al. found that cyanidin-3-galactoside isolated from cranberry fruit extract was capable of scavenging free radicals and inhibiting the oxidation of low-density lipoproteins [20]. Ho et al. performed studies in mice and found that peonidin-3-glucoside reduced the number of the metastases of lung carcinoma cells [21]. Smeriglio et al. found a glycemia-lowering effect of cyanidin-3-glucoside [22].

Seeram et al. found that the anthocyanin fraction in cranberry fruit extracts inhibited inflammatory processes [23], and cranberry fruit extract reduced inflammatory processes in the liver [24]. Santana et al. conducted studies in mice and found that cranberry fruit extract was effective in the treatment of acute pancreatitis [25]. Seeram et al. conducted studies with cancer cell lines. The studies showed that anthocyanins, proanthocyanidins, and flavonol glycosides isolated from cranberry fruit extracts had antiproliferative effects on oral, colon, and prostate cancer cells [26].

Cranberry fruits, both fresh and processed, are used in the production of food supplements, juice, and in the confectionery industry [7,8]. Cranberry fruits and their extracts could be used as natural preservatives, since the biologically active compounds (anthocyanins and phenolic compounds) found in them have antioxidant and antimicrobial effects. Phenolic compounds have antimicrobial activity against bacterial and fungal strains that can cause food spoilage and poisoning [27]. Anthocyanins obtained from cranberry fruit are used as food colorants in confectionery [28]. The ability of anthocyanins to change color from red to blue and other shades depending on the pH of the environment can be used to determine the quality and shelf life of perishable foods and to create smart food packaging [29,30]. The biologically active compounds of cranberry fruit—anthocyanins—can have antimicrobial, antioxidant, and other effects on disease prevention and health in the development of healthy and ecological food products.

The studies of the qualitative and quantitative composition of anthocyanins in cranberry samples are important for the evaluation of the quality of food products, food supplements, and pharmaceuticals. The preparation of high commercial value raw material of cranberry fruit should be based on the evaluation of phytochemical profiles, especially anthocyanins and their distribution in cultivated or wild cranberries.

Determination of anthocyanins content in cranberry fruit are important for the evaluation of fruit samples of small cranberries (*Vaccinium oxycoccos*) and American cranberries (*Vaccinium macrocarpon*) and their cultivars, determining the regularities of the accumulation of biologically active compounds, the optimal time of berry harvesting, and the selection of the most promising cranberry cultivars for introduction.

This study aimed to determine the anthocyanin profiles and antioxidant activity of the fruits of *Vaccinium macrocarpon* cultivars. The performed research will provide new knowledge about the variation in the qualitative and quantitative composition of anthocyanins in cranberry cultivars and is important in determining the most promising cranberry cultivars for cultivation in Lithuanian climatic conditions.

## 2. Materials and Methods

### 2.1. Reagents

Acetonitrile (manufacturer: Sigma-Aldrich, Steinheim, Germany), methanol (manufacturer: Sigma-Aldrich, Steinheim, Germany), formic acid (manufacturer: Merck, Darmstadt, Germany), reference standards for delphinidin-3-galactoside, cyaniding-3-galactoside, cyaniding-3-glucoside, cyaniding-3-arabinoside, peonidin-3-galactoside, peonidin-3-arabinoside, peonidin-3-glucoside, malvidin-3-galactoside, malvidin-3-arabinoside, cyanidin chloride, peonidin chloride, and malvidin chloride were purchased from Extrasynthese (Genay, France). ABTS 2,2′-Azino-bis(3-ethylbenzothiazoline-6-sulfonic acid), Trolox (6-hydroxy-2,5,7,8-tetramethylchroman-2-carboxylic acid), potassium peroxydisulfate, sodium acetate (manufacturer: Scharlau Sentmenat, Barcelona, Spain), ferric (III) chloride hexahydrate (manufacturer: Vaseline-Fabrik Rhenania, Bonn, Germany), TPTZ (2,4,6-Tris(2-pyridyl)-s-triazine) (manufacturer: Carl Roth, (Karlsruhe, Germany), acetic acid (manufacturer: Lach Ner, Neratovice, Czech Republic), hydrochloric acid (manufacturer Sigma-Aldrich, Steinheim, Germany), potassium chloride, and ethanol 96% (*v*/*v*) (manufacturer: AB Stumbras, Kaunas, Lithuania) were also acquired.

### 2.2. Raw Material

**Figure 1:**
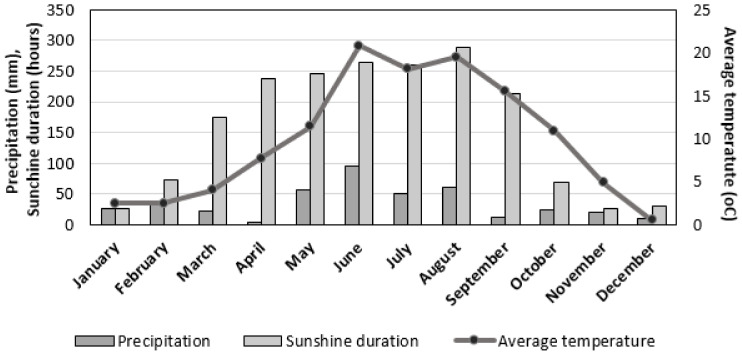
General climate condition (temperature, precipitation, and sunshine duration) in region of Vilnius in 2020.

The fruits examined in the present study were mature and ripe fruit of different cultivars of American cranberry (*Vaccinium macrocarpon* Aiton) (Table 1) grown in Lithuanian climatic conditions, in the collection of the Institute of Botany of the Nature Research Center, Mažieji Gulbinai, Vilnius (54°41′36.6″ N 25°21′56.0″ E). Dynamics of meteorological factors (precipitation (mm), sunshine duration (h), and temperature (°C)) in region of Vilnius in 2020 are presented in Figure 1 [31]. The collection time was September 2020. Cranberry fruits were ground and frozen at −60 °C in an ultra-low-temperature freezer (CVF330/86, ClimasLab SL, Barcelona, Spain). Cranberry fruits were freeze-dried according to the methodology described by Gudžinskaitė et al. [32]. The fruits were powdered in a Retsch GM 200 electric mill (Retsh GmbH, Hahn, Germany). Loss on drying was determined using the method described in the European Pharmacopoeia Ph.Eur.01/2008: 20232 [33].

### 2.3. Preparation of Cranberry Extracts

About 1 g (precise weight) of the lyophilized cranberry powder was extracted with 20 mL of 70% (*v*/*v*) ethanol containing 1% hydrochloric acid in an ultrasonic bath for 15 min at 80 kHz and 565 W at room temperature. The extract was collected and filtered through a membrane filter with a pore size of 0.20 µm. The prepared extracts were stored in dark glass vials at −20 °C.

### 2.4. Spectrophotometric Studies

#### 2.4.1. Determination of Antioxidant Activity

An ABTS∙+ radical cation decolorization assay was applied according to the methodology described by Re et al., 1999 [37] and modified by Raudone et al. [38]. A volume of 3 mL of ABTS^+^ solution (absorbance 0.800 ± 0.02) was mixed with 5 µL of the 5-fold diluted cranberry extract. A decrease in absorbance was measured at a wavelength of 734 nm after keeping the samples for 30 min in the dark. A standard curve (*y* = 0.000008*x* − 0.0909; R^2^ = 0.997) was produced by using standard Trolox solutions of 4000–48,000 μmol/L concentration.

The ferric reducing antioxidant power (FRAP) assay was carried out as described by Raudone et al. [39]. The working FRAP solution included TPTZ (0.01 M dissolved in 0.04 M HCl), FeCl_3_·6H_2_O (0.02 M in water), and an acetate buffer (0.3 M, pH 3.6) at the ratio of 1:1:10. A volume of 3 mL of a freshly prepared FRAP reagent was mixed with 10 µL of the cranberry extract. After 30 min, the absorbance was read at 593 nm using a UV-vis spectrophotometer. A standard curve (*y* = 0.0000166*x* + 0.000950; R^2^ = 0.993) was produced by using standard Trolox solutions of 400–24,000 μmol/L concentration.

#### 2.4.2. Total Anthocyanins Content Determination

The total anthocyanin content was determined according to the pH differential method as described by Lee et al. [40]. During the evaluation, 0.625 mL of cranberry extracts were diluted in 25 mL of two different buffers: 0.025 M potassium chloride (pH = 1.0) and 0.4 M sodium acetate (pH = 4.5). The samples were kept in the dark for 20 to 30 min and then the absorption (A) was measured at λ = 520 nm and λ = 700 nm. The diluted test portions were read versus a blank cell filled with distilled water.

The anthocyanin pigment concentration, expressed as cyanidin-3-glucoside equivalents, was calculated as follows:

Anthocyanin content (mg/L) = A × MW × DF × 1000/(ε × l)

where: A = (A520 − A700)_pH1.0_ − (A520 − A700)_pH4.5_; MW (molecular weight) = 449.2 g mol^−1^ for cyanidin-3-glucoside; DF = dilution factor; l = cuvette path length in cm (1 cm); ε = 26,900 mol extinction coefficient, in L × mol^–1^ × cm^–1^ for cyanidin-3-glucoside.

### 2.5. The UPLC-PDA Method

The analysis of the qualitative and quantitative composition of anthocyanins in cranberry fruit was performed using the UPLC methodology validated by Vilkickyte et al. [19]. The identification of the peaks was performed by comparing the UV absorption spectrum of the reference standard with the UV absorption spectrum of the matrix peaks of American cranberry, using the same retention time.

### 2.6. Statistical Analysis

Data analysis was performed using Microsoft Excel 2016 (Microsoft, Santa Rosa, CA, USA) and SPSS Statistics 27 (IBM, Armonk, NY, USA). All experiments were carried out in triplicate, and presented as the mean value ± SD. Significant differences between samples were determined using ANOVA with Tukey’s test for multiple comparisons. The variability of the results were evaluated using coefficients of variation (CV). Principal component analysis (PCA) and hierarchical cluster analysis applying the between-groups clustering method with Euclidean distances were performed to elucidate the groupings of cranberries. Correlational analysis was performed using Pearson coefficient. Level of significance α = 0.05.

## 3. Results and Discussion

### 3.1. Determination of the Qualitative and Quantitative Composition of Anthocyanins in Cranberry Fruit Samples via the UPLC Method

**Figure 2:**
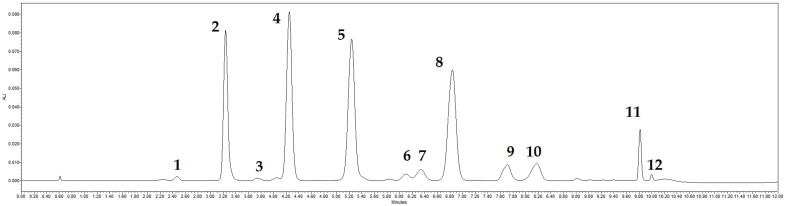
Chromatogram profile of anthocyanins in fruit samples of American cranberries at 520 nm: 1—Delphinidin-3-galactoside, 2—Cyanidin-3-galactoside, 3—Cyanidin-3-glucoside, 4—Cyanidin-3-arabinoside, 5—Peonidin-3-galactoside, 6—Peonidin-3-glucoside, 7—Malvidin-3-galactoside, 8—Peonidin-3-arabinoside, 9—Cyanidin, 10—Malvidin-3-arabinoside, 11—Peonidin, 12—Malvidin.

Increasing consumer awareness creates the demand for the products with health-promoting effects and capabilities of prevention of various pathological processes. To assure the quality of such products, it is important to study the chemical composition of cranberry fruits and to conduct qualitative and quantitative evaluation of their biologically active compounds [41]. The chromatogram of anthocyanins and anthocyanidins in cranberry fruit samples identified via the UPLC-DAD method is shown in Figure 2.

**Figure 3:**
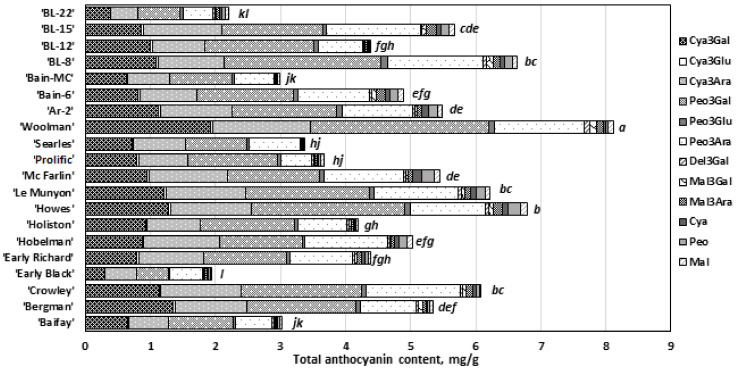
Variation of anthocyanin content in the cranberries of American cultivars. Statistically significant differences between the total anthocyanin content in fruit samples of cranberry cultivars are marked by different letters (*p* < 0.05): Cya3Gal—Cyanidin-3-galactoside, Cya3Glu—Cyanidin-3-glucoside, Cya3Ara—Cyanidin-3-arabinoside, Peo3Gal—Peonidin-3-galactoside, Peo3Glu—Peonidin-3-glucoside, Peo3Ara—Peonidin-3-arabinoside, Del3Gal—Delphinidin-3-galactoside, Mal3Gal—Malvidin-3-galactoside, Mal3Ara—Malvidin-3-arabinoside, Cya—Cyanidin, Peo—Peonidin, Mal—Malvidin.

Four anthocyanin glycosides, namely cyanidin-3-galactoside, cyanidin-3-arabinoside, peonidin-3-galactoside, and peonidin-3-arabinoside, predominated in the studied samples of cranberry cultivars grown in Lithuanian (Figure 2) in a range of 15.12–25.41%, 15.29–25.30%, 15.05–37.98%, and 13.25–25.92%, respectively (Figure 3). They accounted for 91.66 ± 2.79% of the total amount of anthocyanins identified. The qualitative profiles determined in our study are consistent with the results of Česonienė et al. and Viskelis et al. [7,42] and these compounds could be regarded as anthocyanin marker compounds. However, their quantitative profiles were variable. Česonienė et al. have determined that cyanidin-3-galactoside accounted for 24.11%, cyanidin-3-arabinoside for 18.73%, peonidin-3-galactoside for 33.29%, and peonidin-3-arabinoside for 16.7% of total determined anthocyanins [42]. Vikelis et al. have determined the following marker compound composition in fruit samples of ‘Stevens’, ‘Pilgrim’, ‘Ben Lear’, and ‘Black Veil’: cyanidin-3-galactoside—20.5%, cyanidin-3-arabinoside—19%, peonidin-3-galactoside—32.7%, and peonidin-3-arabinoside—6.7% of total anthocyanin content [7]. Furthermore, Zhang et al. have studied the composition of fruit wine of cranberry cultivars ‘Stevens’, ‘Pilgrim’, and ‘Bergman’. The anthocyanin content of cranberry wine has been found to be about 50% cyanidin-3-arabinoside and about 27% peonidin-3-arabinoside. The anthocyanin content in the wine of the fruits of other cranberry cultivars have been determined with different compositions, namely of 4% of cyanidin-3-galactoside, 9% of peonidin-3-galactoside, 1.5% of cyanidin-3-glucoside, and 7% of peonidin-3-glucoside [43].

In our study, the highest amounts of cyanidin-3-galactoside (1.92 ± 0.02 mg/g) were determined in cranberry samples of the ‘Woolman’ cultivar (*p* < 0.05). Statistically significantly lower levels of cyanidin-3-galactoside (1.36 ± 0.03 mg/g, 1.28 ± 0.04 mg/g, and 1.21 ± 0.02 mg/g) were found in fruit samples of cranberry cultivars ‘Bergman’, ‘Howes’, and ‘Le Munyon’, respectively. The lowest cyanidin-3-galctoside levels (0.29 ± 0.01 mg/g and 0.38 ± 0.01 mg/g) were determined in the samples of the ‘Early Black’ cultivar and the ‘BL-22’ genetic clone, respectively (*p* < 0.05).

The highest content of cyanidin-3-arabinoside (1.51 ± 0.01 mg/g) was determined in cranberry samples of the ‘Woolman’ cultivar (*p* < 0.05). Lower levels of cyanidin-3-arabinoside (1.24 ± 0.02 mg/g, 1.25 ± 0.02 mg/g, 1.22 ± 0.01 mg/g, and 1.21 ± 0.02 mg/g) were found in fruit samples of cranberry cultivars ‘Crowley’, ‘Howes’, ‘Le Munyon’, and ‘Mc Farlin’, respectively. The lowest cyanidin-3-arabinoside content (0.40 ± 0.02 mg/g) was found in fruit samples of the ‘BL-22’ genetic clone (*p* < 0.05).

The highest content of peonidin-3-galactoside (2.74 ± 0.03 mg/g) was determined in fruit samples of the ‘Woolman’ cultivar (*p* < 0.05). Statistically significantly lower levels of peonidin-3-galactoside (2.35 ± 0.02 mg/g and 2.41 ± 0.03 mg/g) were determined in cranberry samples of the ‘Howes’ cultivar and the ‘BL-8’ genetic clone, respectively. The lowest content of peonidin-3-galactoside (0.49 ± 0.02 mg/g) was determined in samples of the ‘Early Black’ cultivar (*p* < 0.05).

The highest levels of peonidine-3-arabinoside (1.48 ± 0.02 mg/g, 1.38 ± 0.02 mg/g, and 1.46 ± 0.02 mg/g) were determined in fruit samples of the genetic clone ‘BL-8’ and ‘Woolman’ and ‘Crowley’ cultivars, respectively (*p* < 0.05). The lowest peonidine-3-arabinoside levels (0.50 ± 0.01 mg/g and 0.47 ± 0.02 mg/g) were determined in cranberries of the ‘Early Black’ cultivar and the ‘BL-22’ genetic clone, respectively (*p* < 0.05).

The analysis of the quantitative composition of the anthocyanin complex in cranberry fruit samples showed that aglycones formed a small part of the anthocyanin complex. The highest content of one of the anthocyanidins, cyanidin (0.133 ± 0.01 mg/g), was determined in fruit samples of the ‘McFarlin’ cultivar (*p* < 0.05), while no cyanidin was detected in fruit samples of the ‘Searles’ cultivar. Peonidin content ranged from 0.17% to 3.53% in cranberry fruit samples. The highest levels of peonidin (0.184 ± 0.01 mg/g and 0.192 ± 0.01 mg/g) were found in fruit samples of ‘Howes’ and ‘McFarlin’ cultivars, respectively (*p* < 0.05). The lowest aglycone peonidin content (0.006 ± 0.00 mg/g) was determined in cranberry samples of the ‘Searles’ cultivar (*p* < 0.05). Malvidin content ranged from 0.21% to 2.60% in cranberry samples. The highest content of the anthocyanidin malvidin (0.107 ± 0.01 mg/g) was found in fruit samples of the ‘Howes’ cultivar, and the lowest (0.012 ± 0.00 mg/g) in fruit samples of the ‘Crowley’ cultivar (*p* < 0.05). These results suggest that genotypes have characteristic variation in the amounts of individual anthocyanins and anthocyanidins.

The highest total anthocyanin content (8.13 ± 0.09 mg/g) was determined in cranberry samples of the ‘Woolman’ cultivar (*p* < 0.05). Lower total anthocyanin levels (6.90 ± 0.12 mg/g, 6.22 ± 0.15 mg/g, 6.07 ± 0.08 mg/g, and 6.63 ± 0.11 mg/g) were found in cranberry samples of ‘Howes’, ‘Le Munyon’, and ‘Crowley’ cultivars and in the ‘BL-8’ genetic clone, respectively. The lowest total anthocyanin levels (1.95 ± 0.11 mg/g and 2.21 ± 0.15 mg/g) were found in fruit samples of the ‘Early Black’ cultivar and the ‘BL-22’ genetic clone, respectively (*p* < 0.05).

Gardana et al. have studied the anthocyanin content in fruit samples of ‘Ben Lear’, ‘Howes’, ‘Stevens’, and ‘Bergman’ cultivars and found that the total anthocyanin content ranged from 2.4 mg/g to 5.6 mg/g [44]. The total anthocyanin levels (5.07 mg/g in cranberry samples of the ‘Howes’ cultivar and 3.36 mg/g in cranberry samples of the ‘Bergman’ cultivar) that were found by the authors were lower than those found in our study (6.80 mg/g and 5.34 mg/g in fruit samples of the ‘Howes’ and ‘Bergman’ cultivars, respectively) [44]. Brown et al. have examined the anthocyanin composition in fruit samples of cultivars ‘Ben Lear’, ‘Bergman’, ‘GH 1’, ‘Pilgrims’, and ‘Stevens’ and found that the total anthocyanin content ranged from 2.81 mg/g to 7.98 mg/g [45]. The total anthocyanin content that were found by the authors in fruit samples of the ‘Bergman’ cultivar (7.02 mg/g) were higher than that found in our study (5.34 mg/g) [45]. The total anthocyanin content in samples of fresh cranberry fruit of the ‘Bergman’ and ‘Early Richard’ cultivars (0.48 mg/g and 0.52 mg/g, respectively) were found in a study conducted by Narwojsz et al. to be about 10 times lower than the total anthocyanin levels in the lyophilized raw material of ‘Bergman’ and ‘Early Richard’ cultivars found in our study (4.38 mg/g and 5.34 mg/g, respectively) [46].

The quantified mean amounts of anthocyanins and anthocyanidins in fruit samples of cranberry cultivars can be presented in the following decreasing order: peonidin-3-galactoside > peonidin-3-arabinoside > cyanidin-3-arabinoside > cyanidin-3-galactoside > peonidin > malvidin-3-arabinoside > peonidin-3-glucoside > malvidin > cyanidin > malvidin-3-galactoside > delphinidin-3-galactoside > cyanidin-3-glucoside. The chromatographic profile of cranberry anthocyanins is characteristic, and thus its identification can be applied for establishing the authenticity of the cranberry plant raw material and identifying possible falsifications with other anthocyanin-accumulating botanical raw materials [47].

### 3.2. Quantification of the Total Anthocyanin Content in Cranberry Samples

Spectrophotometric analysis is used to evaluate the quality of herbal raw materials and their preparations. This method is used to assess the quantitative composition of the groups of biologically active compounds. The spectrophotometric pH differential method is simple, fast, and economical. This method is often used in practice to determine the total anthocyanin content in a sample [40,48,49]. The total anthocyanin content determined via the spectrophotometric pH differential method should be evaluated individually for each plant raw material, as the test results are influenced by the methodology used [50]. The total amount of anthocyanins is usually expressed in CGE (cyanidin-3-glucoside equivalent), as cyanidin-3-glucoside is the predominant anthocyanin in many fruits and vegetables, yet it does not necessarily reflect the anthocyanin composition of the raw material under study [51,52].

The quantitative composition of anthocyanins determined by pH differential method were analyzed in comparison with the total identified anthocyanin origin compounds determined by the ultra-high performance liquid chromatography method (Table 2).

The total anthocyanin content determined in the studied fruit samples of American cranberries via the pH differential method ranged from 1.13 ± 0.02 mg CGE/g to 5.09 ± 0.24 mg CGE/g. The lowest total anthocyanin content (1.13 ± 0.02 mg CGE/g) was found in fruit samples of the ‘Early Black’ cultivar, and the maximum total content of anthocyanins (5.09 ± 0.24 mg) was found in fruit samples of the ‘Woolman’ cultivar. The total anthocyanin content in the fruit samples of American cranberry cultivars ‘Bergman’, ‘Crowley’, ‘Howes’, ‘Le Munyon’, and ‘McFarlin’ grown in New Zealand’s climatic conditions ranged from 1.34 mg CGE/g to 1.90 mg CGE/g and were about 1.5 times lower than the total anthocyanin content found in our study in samples of the abovementioned cultivars [53].

The total anthocyanin content in cranberry fruit samples determined via the pH differential method was about 1.6 times lower than the total anthocyanin content determined via the UPLC method. The coefficient of variation of the total anthocyanin content in cranberry fruit samples determined via the UPLC method was 33.57%, while that determined via the pH differential method was 37.45%.

Grace et al. in their study have found that the total anthocyanin content (expressed as CGE) in American cranberry fruit samples detected via the application of the pH differential method was 1.4 times lower than that detected via the HPLC method [54]. Lee et al. have found no statistically significant difference between the anthocyanin content in cranberry samples determined via the pH differential method (1.95 ± 0.14 mg CGE/g) and the UPLC method (2.06 ± 0.26 mg CGE/g) [55]. The authors did not find any differences between the compared methods because the total amount of anthocyanins was calculated based on the four predominant anthocyanins, namely cyanidin-3-galactoside, cyanidin-3-arabinoside, peonidin-3-galactoside, and peonidin-3-arabinoside, whereas in our study, the amount of other anthocyanins detected in cranberry fruit samples ranged from 3.71% to 13.23% [55].

The results obtained using pH differential methods and UPLC were highly corresponding and correlated (R = 0.975, *p* < 0.05). A very strong significant correlation between the total anthocyanin content in cranberry fruit samples assessed via the pH differential method and the UPLC method was also found in other studies, the correlation coefficients being r = 0.98, r = 0.925, and r ≥ 0.99 (*p* < 0.05) [50,54,55]. The very strong correlation between the pH differential method and the UPLC method indicates that the anthocyanin content determined by both methods is similar, but the results obtained should be evaluated on a case-by-case basis, depending on the raw material and the nature of the study.

### 3.3. Hierarchical Cluster Analysis and Principal Component Analysis of the Distribution of Anthocyanin Content in Fruit Samples of American Cranberry

**Figure 4:**
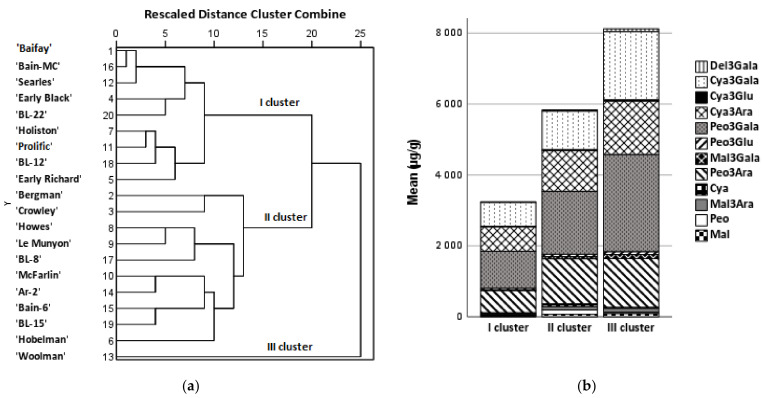
A dendrogram of the distribution of American cranberry cultivars into similar clusters according to the anthocyanin content in fruit samples (**a**); a diagram of the distribution of mean anthocyanin content in clusters (**b**). Cya3Gala—Cyanidin-3-galactoside, Cya3Glu—Cyanidin-3-glucoside, Cya3Ara—Cyanidin-3-arabinoside, Peo3Gala—Peonidin-3-galactoside, Peo3Glu—Peonidin-3-glucoside, Peo3Ara—Peonidin-3-arabinoside, Del3Gala—Delphinidin-3-galactoside, Mal3Gala—Malvidin-3-galactoside, Mal3Ara—Malvidin-3-arabinoside, Cya—Cyanidin, Peo—Peonidin, Mal—Malvidin.

The analysis of the similarity of the composition of anthocyanins in American cranberry cultivars introduced and grown in Lithuanian climatic conditions was based on the quantitative composition of anthocyanins determined in the samples of different cultivars and was carried out by performing a hierarchical cluster and principal component analysis. The cluster analysis of the samples of different cultivars of American cranberry was performed on the basis of the quantitative composition of anthocyanins delphinidin-3-galactoside, cyanidin-3-galactoside, cyanidin-3-glucoside, cyanidin-3-arabinoside, peonidine-3-galactoside, peonidin-3-glucoside, peonidin-3-galactoside, peonidine-3-arabinoside, cyanidin, malvidin-3-arabinoside, peonidin, and malvidin. Fruit samples of cranberry cultivars were divided into three clusters (Figure 4).

Fruit samples of cranberry cultivars ‘Baifay’, ‘Early Black’, ‘Early Richard’, ‘Holiston’, ‘Prolific’, ‘Searles’, ‘Bain-6’, ‘BL-12’, and ‘BL-22’ were assigned to cluster I because their total anthocyanin content was by 1.7 times lower than the average total anthocyanin content. The total amount of anthocyanins found in the fruit samples of the cluster II cultivars (‘Bergman’ ‘Crowley’, ‘Hobelman’, ‘Howes’, ‘Le Munyon’, ‘Mc Farlin’, ‘Ar-2’, ‘Bain-MC’, ‘BL-8’, and ‘BL-15’) was close to the mean anthocyanin content. Cluster II is considered to consist of cranberry cultivar samples which have higher than average levels of anthocyanidins (cyanidin, peonidin, and malvidin). Cluster III consisted of one American cranberry cultivar ‘Woolman’. The total anthocyanin content in cranberry fruit samples of the ‘Woolman’ cultivar was twice the mean total anthocyanin content in the studied cultivars.

**Figure 5:**
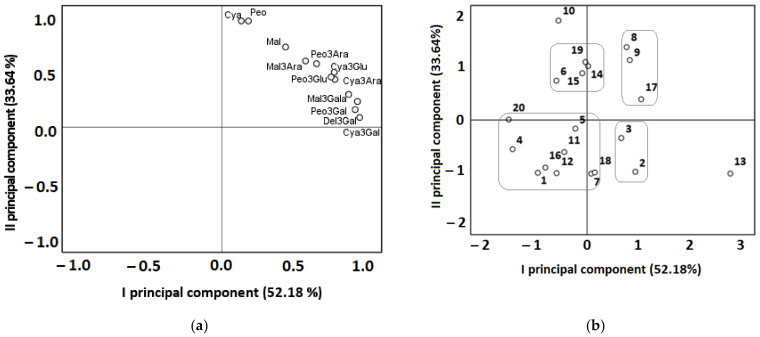
Principal component analysis loading (**a**) and score (**b**) plots of different cranberry fruit samples: (**a**) Cya3Gala—Cyanidin-3-galactoside, Cya3Glu—Cyanidin-3-glucoside, Cya3Ara—Cyanidin-3-arabinoside, Peo3Gal—Peonidin-3-galactoside, Peo3Glu—Peonidin-3-glucoside, Peo3Ara—Peonidin-3-arabinoside, Del3Gal—Delphinidin-3-galactoside, Mal3Gala—Malvidin-3-galactoside, Mal3Ara—Malvidin-3-arabinoside, Cya—Cyanidin, Peo—Peonidin, Mal—Malvidin; (**b**) 1—‘Baifay’, 2—‘Bergman’, 3—‘Crowley’, 4—‘Early Black’, 5—‘Early Richard’, 6—‘Hobelman’, 7—‘Holiston’, 8—‘Howes’, 9—‘Le Munyon’, 10—‘Mc Farlin’, 11—‘Prolific’, 12—‘Searles’, 13—‘Woolman’, 14—‘Ar-2’, 15—‘Bain-MC’, 16—‘Bain-6’, 17—‘BL-8’, 18—‘BL-12’, 19—‘BL-15’, 20—‘BL-22’.

A principal component analysis (PCA) was performed to evaluate the quantitative variation of the identified anthocyanin group compounds in different American cranberry cultivars (Figure 5). Two main components were used for the analysis, explaining 85.82% of the total data variance. The first component (PC I), which explained 52.18% of the total data variance, had a significant correlation with the anthocyanin galactosides cyanidin-3-galactoside (0.958), peonidin-3-galactoside (0.942), delphinidin-3-galactoside (0.926), and malvidin-3-galactoside (0.881), and a strong positive correlation with cyanidin-3-arabinoside (0.786), cyanidin-3-glucoside (0.781), peonidin-3-glucoside (0.759), and peonidin-3-arabinoside (0.658). The second component (PC II), which explained 33.64% of the total data variance, had a significant correlation with cyanidin (0.973) and peonidin (0.973), and a positive correlation with malvidin (0.738) and malvidin-3-arabinoside (0.613).

American cranberry cultivars located in the negative squares of PC I and PC II (‘Baifay’, ‘Early Black’, ‘Early Richard’, ‘Holiston’, ‘Prolific’, ‘Searles’, ‘Bain-6’, ‘BL-12’, and ‘BL-22’) coincided with cluster I in the cluster analysis, which had a lower-than-average total anthocyanin content. The cranberry cultivars ‘Bergman’ and ‘Crowley’ formed the second group of similar cultivars with above-average total anthocyanin content and below-average total anthocyanidin (cyanidin, peonidin, and malvidin) content. The total anthocyanin content in the samples of the hybrid cultivars ‘Bergman’ (a hybrid of ‘Early Black’ and ‘Searles’) and ‘Crowley’ (a hybrid of ‘Mc Farlin’ and ‘Prolific’) was higher than that in samples of the precursor cultivars ‘Early Black’ and ‘Searles’, and ‘Mc Farlin’ and ‘Prolific’, respectively. Diaz-Garcia et al. found that fruit samples of the second- and third-generation cycle cultivars contained higher levels of anthocyanins than the samples of the early selection attempts did [56].

The American cranberry cultivar ‘Woolman’ was far removed from other cranberry cultivars in the PCA chart because the total anthocyanin content found in it was almost two times higher than the mean total anthocyanin content. Exceptionally high levels of cyanidin-3-galactoside and peonidin-3-galactoside were determined in samples of the cranberry cultivar ‘Woolman’, but the total content of anthocyanidins (cyanidin, peonidin, and malvidin) was lower than their mean total content in cultivar samples. In our previously published study, samples of the cranberry cultivar ‘Woolman’ differed from samples of cultivars ‘Baiwfay’, ‘Drever’, ‘Bain’, ‘Bergman’, ‘Searles’, ‘Holliston’, and ‘Piligrim’ in that they contained high levels of phenolic acids and dihydrochalcones and low levels of flavonols and flavan-3-ols [32].

The total content of anthocyanidin galactosides in fruit samples of the group consisting of the cranberry cultivar ‘Hobelman’ and genetic clones ‘Ar-2’, ‘Bain-MC’, and ‘BL-15’ was close to the mean total amount of galactosides in the studied cultivars, and the total content of anthocyanidins (cyanidin, peonidin, and malvidin) was higher than the mean total amount of anthocyanidins. The cranberry cultivar ‘Mc Farlin’ was far from other cultivars in the PCA chart because its fruit samples contained two times the average amount of aglycones (cyanidin, peonidin, and malvidin). The group of American cranberry cultivars ‘Howes’, ‘Le Munyon’, and ‘BL-8’ located in the positive quadrant of PC I and PC II was characterized by higher anthocyanidin galactoside and total anthocyanidin contents compared to the mean total anthocyanidin and galactoside contents.

### 3.4. Determination of Antioxidant Activity

Recently, many studies have been conducted on phenolic compounds in plant matrices as natural antioxidants with protective antioxidant properties as well as on the application of these phenolic compounds for prophylactic purposes [57]. The antioxidant effects of anthocyanins occur through a number of complex mechanisms by which anthocyanins can directly scavenge free radicals, prevent the formation of reactive oxygen species (by forming chelating compounds with metals, they inhibit redox reactions and inhibit xanthine oxidase and NADPH oxidase), or promote the release of antioxidant enzymes [58,59]. The antioxidant properties of phenolic compounds are associated with biological effects such as anti-inflammatory, anticancer, and antimicrobial effects. In vitro assays for antiradical and reductive activity are expedient to determine the potency of the potential antioxidant activity of the extracts of fruit samples of the tested cultivars.

**Figure 6:**
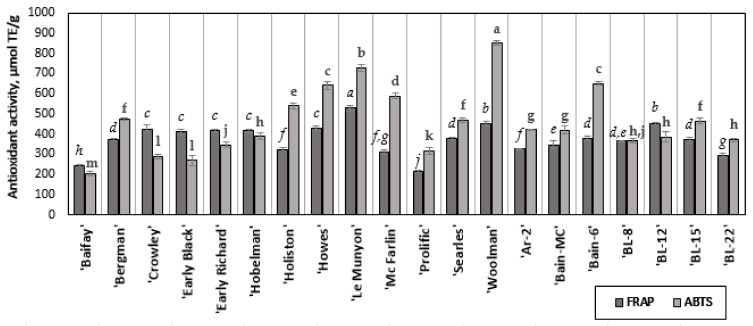
Determination of the antioxidant activity of cranberry extracts. The letters of different fonts indicate statistically significant differences in the antioxidant activity of the fruit extracts of cranberry cultivars determined by the FRAP and ABTS methods (*p* < 0.05).

The strongest antiradical activity detected by using the ABTS method (849.75 ± 10.88 µmol TE/g) was found in samples of the cranberry cultivar ‘Woolman’ (Figure 6). The strongest antioxidant activity determined by using the FRAP method (528.05 ± 12.16 µmol TE/g) was determined in samples of the cranberry cultivar ‘Le Munyon’. The weakest antiradical activity detected by applying the ABTS method (203.20 ± 9.19 µmol TE/g) was determined in samples of the cranberry cultivar ‘Baifay’. The weakest reducing activity (215.23 ± 3.24 µmol TE/g) was found in fruit samples of the ‘Prolific’ cultivar.

Oszmiański et al. have investigated the antioxidant activity of fruit extracts of ‘Howes’ by applying the ABTS and FRAP methods [5]. However, in our study, the reducing and radical scavenging activities of ‘Howes’ fruit extracts were 4-fold and 3-fold greater, respectively.

The correlation between the total anthocyanin content and antioxidant capacity was determined for the ABTS method (r = 0.635; *p* < 0.001), and for the FRAP method (r = 0.507; *p* < 0.001). Oszmiański et al. determined similar correlation coefficients for the ABTS (r = 0.675), FRAP (r = 0.614), and DPPH (r = 0.602) methods. Chaves et al. have found that the total anthocyanin content determined in the samples of blackberries, blueberries, and strawberries correlated with the antiradical capacity of the extracts determined by the DPPH and ABTS methods (respectively, r = 0.86, r = 0.82) [60]. The positive correlation of anthocyanin content with antioxidant activity suggests that these compounds may contribute to the antioxidant effects of fruit extracts. The American cranberries, besides the great fraction of anthocyanins, also contain other phenolic origin compounds such as flavonoids, phenolic acids, and proanthocyanidins [47]. The anthocyanins together with other phenolic compounds present in the extracts express the total antioxidant activity of the extract.

Scientific studies confirm that antioxidant activity mechanisms are interrelated with the biological activities expressed in the body and substantiates that the bioavailability of phenolic compounds is highly important for their bioactivities in vivo [61]. The data in the literature show that, after ingestion, anthocyanins are quickly absorbed through the stomach and the small intestine by various mechanisms [62]. Nevertheless, small amounts of anthocyanins are absorbed, and plasma levels of cranberry anthocyanins appear to be too low to compete effectively with antioxidants such as ascorbate and glutathione [63]. However, the unabsorbed part of anthocyanins reach the colon, where they can modulate the microbiota [62,63]. Consumption of anthocyanins can modify the colonization of the gut microbiota by the stimulation of the growth of beneficial bacteria, such as *Bifidobaterium* spp. and *Lactobacillus* spp., and the inhibition the growth of pathogenic bacteria, such as *Staphylococcus aureus* and *Salmonella typhimurium* [62,64]. Gutpaper et al. showed that obese mice with a diet of 200 mg/kg cranberry extract per 8 weeks increased the *Akkermansia* population and induced features of the prevention of metabolic phenotypes linked to obesity. Furthermore, treatment with cranberry extract was found to lower content of intestinal triglyceride and to reduce inflammation and oxidative stress of the intestinal tract [65]. The beneficial effects of anthocyanins could be related to the effects on the intestinal tract, which are important for various biochemical processes in the body.

## 4. Conclusions

Cranberry’s phytochemical profile is genotype-dependent and this study provides new knowledge on the variation in the composition of anthocyanins and anthocyanidins in different cranberry cultivars. Moreover, this study allows for the assessment of the quality of cranberry fruit and the use of high-quality cranberry raw material for food and health promotion. Four anthocyanin compounds predominated in fruit samples of American cranberry cultivars: cyanidin-3-galactoside, cyanidin-3-arabinoside, peonidin-3-galactoside, and peonidin-3-arabinoside, and they can be regarded as qualitative profile markers of American cranberry products. The chromatographic profiles are species-characteristic and can be applied for cranberry authenticity studies.

The fruits of American cranberry cultivars ‘Woolman’ and ‘Le Munyon’ distinguished with the highest content of anthocyanins, and their extracts had the strongest antioxidant activity. The cultivation of cranberry cultivars ‘Woolman’, ‘Howes’, and ‘Le Munyon’ in Lithuanian climatic conditions could be carried out in gardens and collections. The results of this study allow for evaluating the qualitative and quantitative composition of anthocyanins and anthocyanidins in fruit samples of the grown cultivars and for ensuring the preparation of high-quality cranberry raw material. High-quality phytochemically characterized American cranberry extracts rich in natural antioxidants can be used in the production of high-added-value products, food supplements, and other functional ingredients for nutrition and health.

## Figures and Tables

**Table 1:** Characteristics typical of American cranberry cultivars; ND (no data).

No.	Cranberry Cultivar	Country of Origin and Year	Cultivar Characteristics	Reference
1	‘Baifay’	ND	ND	
2	‘Bergman’	1961	‘Early Black’ × ‘Searles’ hybrid, abundantly growing, medium-early, fertile. The berries are suitable for storage.	[4,8]
3	‘Crowley’	1961	‘McFarlin’ × ‘Prolific’ hybrid. The berries are of medium size, dark red, suitable for processing.	[4,8]
4	‘Early Black’	1835, MA, USA	Native Selection. The berries are of medium size, sometimes small, blackish red. It grows abundantly and is moderately fertile.	[4,8,34]
5	‘Early Richard’	1870, NJ, USA	A medium early cultivar. Medium-sized, dark red berries. Grows abundantly. The berries are not very suitable for storage.	[8,35]
6	‘Habelman’	ND	ND	
7	‘Holliston’	1885, MA, USA	Native Selection, a medium-early cultivar.	[36]
8	‘Howes’	1843, MA, USA	Native Selection. A late cultivar, disease resistant, fertile. The berries are small, medium-sized, store a lot of pectin, are suitable for storage, and are resistant to frost.	[4,8,34]
9	‘Le Munyon’	1960, NJ, USA	Native Selection, a very early fertile cultivar with medium-sized large dark red berries.	[4,8,34]
10	‘McFarlin’	1874, MA, USA	Native Selection.	[4,34]
11	‘Prolific’	1900, MI, USA	Native Selection.	[4,34]
12	‘Searles’	1893, WI, USA	Native Selection. A medium-early cultivar. The berries are large, red, not suitable for storage. The cultivar is fertile, with high matching stems.	[4,8,34]
13	‘Woolman’	1897, NJ, USA	An early cultivar.	[8,35]
14	‘Ar-2’	ND	A late cultivar.	[8]
15	‘Bain-6’	WI, USA	Native Selection.	[36]
16	‘Bain-MC’	ND	A late cultivar.	[8]
17	‘BL-8’	ND	Is characterized by early berry ripening, lush growth, and medium-sized, large, dark red, oval berries.	[8]
18	‘BL-12’	ND	ND	
19	‘BL-15’	ND	Is characterized by early berry ripening, lush growth, and medium-sized, large, dark red, oval berries.	[8]
20	‘BL-22’	ND	ND	

**Table 2:** Total anthocyanin content in fruit samples of American cranberry cultivars determined via UPLC and the spectrophotometric pH differential methods.

Cultivar	UPLC mg/g	pH Differential Method
‘Baifay’	3.02 ± 0.052	1.54 ± 0.018
‘Bergman’	5.34 ± 0.076	3.36 ± 0.137
‘Crowley’	6.07 ± 0.057	3.72 ± 0.050
‘Early Black’	1.95 ± 0.110	1.13 ± 0.021
‘Early Richard’	4.38 ± 0.053	2.67 ± 0.145
Hobelman’	5.03 ± 0.002	3.10 ± 0.123
‘Holiston’	4.19 ± 0.030	2.30 ± 0.066
‘Howes’	6.79 ± 0.106	4.37 ± 0.101
‘Le Munyon’	6.22 ± 0.077	3.60 ± 0.149
‘Mc Farlin’	5.44 ± 0.026	3.41 ± 0.116
‘Prolific’	3.66 ± 0.002	1.90 ± 0.089
‘Searles’	3.37 ± 0.001	1.86 ± 0.103
‘Woolman’	8.13 ± 0.093	5.09 ± 0.244
‘Ar-2’	5.49 ± 0.010	3.18 ± 0.010
‘Bain-6’	4.89 ± 0.079	2.88 ± 0.101
‘Bain-MC’	2.99 ± 0.016	1.53 ± 0.073
‘BL-8’	6.63 ± 0.070	3.88 ± 0.030
‘BL-12’	4.39 ± 0.001	2.53 ± 0.078
‘BL-15’	5.67 ± 0.073	3.57 ± 0.244
‘BL-22’	2.21 ± 0.088	1.41 ± 0.002

## Data Availability

All datasets generated for this study are included in the article.
